# A Survey of 3D Indoor Localization Systems and Technologies

**DOI:** 10.3390/s22239380

**Published:** 2022-12-01

**Authors:** Andrey Sesyuk, Stelios Ioannou, Marios Raspopoulos

**Affiliations:** 1 School of Engineering, University of Central Lancashire, Preston PR12HE, UK; 2 School of Sciences, University of Central Lancashire Cyprus, Larnaca 7080, Cyprus

**Keywords:** 3D indoor localization, location-based services, Internet of Things

## Abstract

Indoor localization has recently and significantly attracted the interest of the research community mainly due to the fact that Global Navigation Satellite Systems (GNSSs) typically fail in indoor environments. In the last couple of decades, there have been several works reported in the literature that attempt to tackle the indoor localization problem. However, most of this work is focused solely on two-dimensional (2D) localization, while very few papers consider three dimensions (3D). There is also a noticeable lack of survey papers focusing on 3D indoor localization; hence, in this paper, we aim to carry out a survey and provide a detailed critical review of the current state of the art concerning 3D indoor localization including geometric approaches such as angle of arrival (AoA), time of arrival (ToA), time difference of arrival (TDoA), fingerprinting approaches based on Received Signal Strength (RSS), Channel State Information (CSI), Magnetic Field (MF) and Fine Time Measurement (FTM), as well as fusion-based and hybrid-positioning techniques. We provide a variety of technologies, with a focus on wireless technologies that may be utilized for 3D indoor localization such as WiFi, Bluetooth, UWB, mmWave, visible light and sound-based technologies. We critically analyze the advantages and disadvantages of each approach/technology in 3D localization.

## 1. Introduction

For centuries, scientists have been fascinated by the idea of determining position. The first positioning systems appeared several millennia ago, when people driven by their need to know their position when travelling typically used natural landmarks to orientate themselves before establishing their own landmarks (trails, lighthouses, etc). Down the line, other approaches were introduced such as celestial and astronomic methods as well as dead reckoning for ocean navigation. Most of them, however, were extremely limited in range while all of them relied on visual observations, at least to some extent, and hence required clear lines of sight between the light source and the user to be positioned. This restricted their use to specific times of day or to specific weather conditions. The late-nineteenth-century discovery of radio waves paved the way for radio-based navigation/positioning. Radio frequency signals have a greater transmission range than visible light while the can be transmitted through clouds or fog or even propagate as ground waves over vast distances, depending on the frequency of transmission overcoming the range issue for ground-based and satellite-based navigation systems [1].

For many years, location-based services (LBSs), applications and systems have been playing an important role in our lives. Outdoor localization has been very successfully implemented using Global Navigation Satellite Systems (GNSSs) which was typically the de facto approach in wireless positioning. Various GNSSs have been established over the years such as the American Global Positioning System (GPS), Russia’s Global Navigation Satellite System (GLONASS) and the European GALILEO. GNSSs require at least three satellites to determine the specific location on the globe as well as one more satellite for time synchronization. Therefore, it is imperative that these satellites have an unobstructed path between them and the receiving device being positioned. Due to this, heavily shadowed urban areas (areas of dense and tall buildings, usually referred to as ‘urban canyons’) or indoor areas cannot be reliably supported by GNSSs. Therefore, there has been significant work reported in the literature [2] over the last 20-30 years which includes many solutions and approaches for solving the localization problem in satellite-denied environments using—over the years—the current available radio technologies. However, none of these solutions have been standardized as the universal solution (like GNSSs for outdoors) for this kind of environment. Various reasons could be found for this, such as the incremental need for more and more accuracy, the rapid evolution of wireless (and other) technologies that facilitate the support of this higher accuracy which makes the adoption of one system unreasonable if it is going to become obsolete in the near future, the cost and maturity of the underlying technologies to be integrated in mobile devices, etc. Several attempts have been proposed in the literature for improving GNSS localization by fusing the data with IMU sensors and although the accuracy as shown in [3] was indeed improved by 20% or as shown in [4] by 38%, no considerable efforts have been identified that present accurate enough results for indoor environments. Moreover, localization accuracy is relatively subject to the application used. For instance, typical GPS-level accuracy (3-10m) would be sufficient for automobile navigation while room-level accuracy (2-4m) would be enough to identify the presence of someone in a room or area of an indoor environment [5,6].

The global indoor positioning and indoor navigation market was valued at USD 6.1 billion in 2020 and is expected to increase at a compound annual growth rate (CAGR) of 22.9 percent from 2020 to 2025, reaching USD 17.0 billion by 2025 [7]. The growing integration of beacons in cameras, LED lightings, Point of Sale (PoS) devices and digital signage; the proliferation of smartphones, connected devices and location-based apps among customers; and the inefficiency of GPS technology in the indoor environment are driving the global adoption of the indoor location market [8]. The COVID-19 pandemic, which started in 2019 has had an impact on the indoor location market; however, businesses are now using it for facility management, virus monitoring, personnel tracking and management and smart quarantining. Indoor location solutions are being adopted by governments and private organizations across industries to keep residents indoors and track them. For example, Inpixon is providing its location-based technology applications and services free of charge or at a reduced rate (depending on the solution) to healthcare providers and other organizations looking for solutions to help control the spread of COVID-19 or manage the impact of the pandemic to ensure citizens’ safety and well being [9,10].

**Figure 1:**
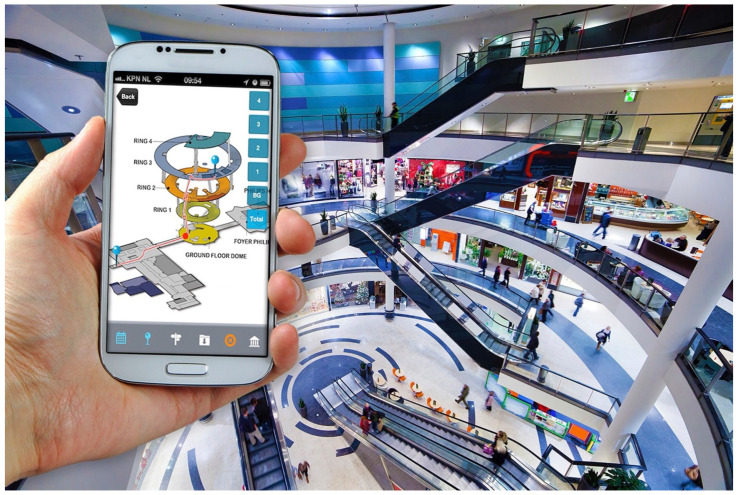
3D indoor positioning application example in a multi-storey mall (by Unknown Author (https://meet.bnext.com.tw/blog/view/3442? accessed on 26 November 2022)— is licensed under CC BY-NC-ND).

In the last couple of decades, there have been several positioning systems proposed and implemented using different techniques and approaches in an attempt to tackle the indoor localization problem. Most such systems solve the problem only in two dimensions, meaning that the position is estimated only on a horizontal (x-y) plane, ignoring the vertical (z) dimension. One practical implication of this could be the inability to recognize if a device is located in a pocket or is held up high or whether a user is located on the first or ground floor of a shopping mall (see Figure 1). This additional localization data in some cases might be crucial. Examples may include a drone used for seeding and fertilizing crops in a greenhouse, where knowing the altitude of the drone with respect to the crops is important or a drone used in search and rescue operations to rescue climbers in canyons or miners in mines, where GNSSs might fail. In most of these cases, accuracy better than sub-meter level is required to avoid crashing the UAV on obstacles. Precise 3D positioning can also find applications in supporting wireless communication and effectiveness in antenna orientation and beamforming [11], pilot assignment [12], channel prediction and resource allocation [13]. Furthermore, due to the rapid increase in the world’s population, not only are buildings nowadays built upwards (skyscrapers), but also road traffic in cities is increasing, which will eventually lead to development of self-driving underground cities in the form of tunnels where GPS will no longer be able to provide localization and navigation. The fact that 3D positioning methods enable the identification of the accurate position of UAVs in space, for example, in urban canyon scenarios could also be extended to perform accurate positioning of a device underwater by utilizing more appropriate ranging technologies (e.g., acoustic) [14,15,16]. In the past decade, there has been a tremendous technical development in indoor positioning/navigation; however, there is yet to be technology that is affordable enough for general market adoption, as opposed to outdoor, well established GNSSs. There are so many factors that could play a role in improving localization accuracy, such as signal attenuation, NLOS conditions and even corporal shade, that the precision of the indoor positioning systems is highly vital in order to reach the most accurate results. While there are several papers on 2D indoor positioning in the literature, to the best of our knowledge, no comprehensive survey on 3D indoor positioning has been conducted. Therefore, in this paper, we discuss existing techniques and technologies for 3D indoor localization and establish a precedent for the need of 3D positioning in the said domain. Furthermore, our work follows an intuitive flow by highlighting the challenges and issues in indoor localization and outlining the existing solutions. The utilization of 5G-related technology has become the development trend of the future 3D indoor positioning. 5G operates through MIMO (Multi-user Multiple Input Multiple Output) antennas, which provide a precise orientation of the signal in one specific direction instead of a multi-directional broadcast. 5G technologies can achieve centimeter-level accuracy for 3D indoor positioning; however, they have not yet reached the necessary global implementation levels. With the rapid rise of more 5G-supported devices, this is soon to be changed. Already, the discussions for the next generation (6G) of wireless systems have begun, envisioning precise localization and sensing systems, as it is believed that 6G systems will accelerate the transition to even higher frequency operation, such as mmWave and THz ranges, as well as significantly wider bandwidths. It is evident that 6G communication opens up a new range of challenges and opportunities in localization and sensing which the authors of [17] summarize in five key research questions: (1) How can cm-level 3D positioning/sensing accuracy be achieved by utilizing the range of technologies used in 6G? (2) How can novel waveform designs be devised to better facilitate localization and sensing in addition to providing the fundamental communication benefits? (3) How can energy efficiency, high positioning/sensing accuracy and (we also say) low cost be supported in very high frequency and very highly mobile and dynamic environments in 6G systems? (4) Can real-time energy efficient AI/ML algorithms be used to further facilitate and support the localization and sensing process? (5) How can the quality and accuracy between active and passive sensing be bridged?

This survey paper focused on studies performed specifically on three-dimensional indoor positioning systems as well as the techniques and technologies to facilitate them by studying various books, articles and papers published by various reputable journals. Although this paper focuses mainly on indoor positioning, some sections may include outdoor positioning examples such as drone navigation as similar principles apply to both cases as both cannot accurately predict the vertical positioning using satellites. Furthermore, this paper excludes two-dimensional indoor positioning studies as there are plenty already reported in the literature.

The remainder of this paper is organized as follows:

- Section 2: We discuss different 3D localization techniques such as geometric approaches like AoA, ToA and TDoA. Moreover, we discuss fingerprinting approaches as they are one of the widely used methods based on metrics such as RSS, CSI, MF and FTM. Furthermore, we discuss the principles of sensor fusion and specifically filtering approaches such as Kalman and Particle Filtering as well as cooperative positioning and PDR. To conclude this section, we discuss the fusion of positioning approaches, also known as hybrid positioning systems and existing systems found in the literature.
- Section 3: We provide a variety of technologies, with a focus on wireless technologies that may be utilized for 3D indoor localization such as WiFi, Bluetooth, UWB, mmWave, visible light and sound-based technologies such as acoustic signals and ultrasound. We analyze the advantages and disadvantages of each technology item primarily focusing the discussion on their applicability for 3D localization.
- Section 4: We discuss the principles of machine learning for 3D indoor localization and provide various existing systems reported to date in the literature.
- Section 5: We provide a critical discussion and conclusions concerning the survey.

A summary of the various notations and symbols used in this paper is shown in Table 1.

## 2. 3D Localization Techniques

In this section, the current state of the art on 3D localization is reviewed. This review covers geometric, fingerprinting-based, sensor-fusion-based as well as hybrid approaches, critically evaluating and quantifying their 3D positioning performance based on the work reported in the literature. At the end of this section in Table 2, a summary of all geometric approaches can be found, describing their advantages and disadvantages, as well as their accuracies found in the literature.

### 2.1. Geometric Approach

**Figure 2:**
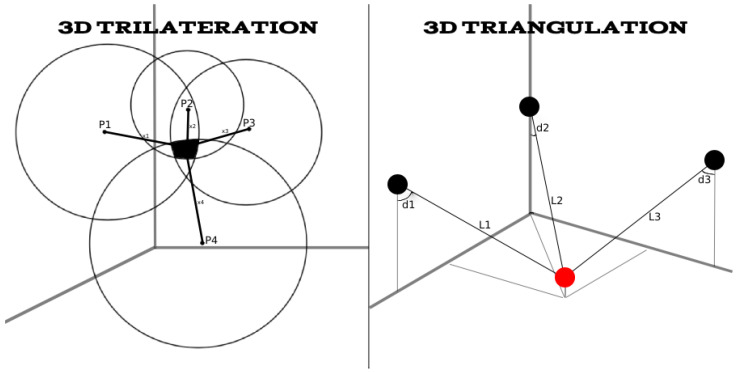
3D Trilateration and 3D Triangulation.

Among the many indoor positioning techniques (2D and 3D) that have been reported in literature, the most widely used and recognized are the ones based on a geometric approach. This approach suggests that localization is generally carried out in two steps. The mobile device first records one or more signal parameters that are dependent on the mobile user’s location from an adequate number of transmitters and then computes the relative location coordinates in a 2D or 3D plane using standard geometry. In this method, there are three approaches: angle-distance, timing-based techniques (ToA and TDoA) and angular-based techniques (AoA). In timing-based techniques the approximate distance to each transmitter is computed by determining the time required for the signal to reach the terminal when transmitted from a specific access point. The latter technique relies on the ability of the terminal to record the angle of arrival of a signal from a given access point or base station. Modern radio technologies such as UWB and even more millimeter-wave (mmWave) radio create opportunities for very accurately estimating the time and angle of arrival (using phased antenna arrays) [18,19,20]. There are three prevalent terminologies that describe the geometric approach to determine position, based on distance or angle of arrival measurements: triangulation, trilateration and angulation (see Figure 2). Triangulation is the estimation of a 2D or 3D location using unilateral or multilateral measurements (the position is determined from the measured lengths of three sides of a triangle). Trilateration is the estimation of location using several distance measurements, whereas angulation uses angles relative to known positions. In this subsection, we will describe techniques which utilize all these approaches [21].

#### 2.1.1. Angle-Distance

The simplest geometrical method for estimating the device’s position is one that uses the distance and angle of arrival from a single transmitter. This appears to be dependent on the device’s ability to execute direction finding and distance measurement. Direction finding on the terminal can be achieved through the use of a rotating directional antenna installed on the mobile terminal or through the use of specific procedures if the system is Multiple Input Multiple Output (MIMO). Ranging can be estimated by either translating the recorded time of arrival into distance (multiplying by the speed of light) or by applying the free space path loss formulation to the recorded received signal strength.

#### 2.1.2. Angle of Arrival—AoA

AoA utilizes the triangulation concept described earlier, where a mobile terminal (MT) obtains the angles of arrival of two signals from two fixed transmitting locations. The main advantage of AoA is that, with it, it is possible to establish a position with as low as two sensors for 2D or three for 3D localization, as there is no need for an extra sensor for time synchronization (as is the case in time-based approaches which lead to distance estimation) [22]. Although AoA can give accurate estimates when the distance between the transmitter and the receiver is modest as compared to RSS approaches, it requires more sophisticated equipment and much more careful calibration and as the transmitter-receiver distance increases, its accuracy decays, meaning that a small error in the angle of arrival calculation translates into a large error in the actual location estimation. Furthermore, because of multi-path effects in indoor environments, the AoA may be sometimes difficult to measure [2].

The estimation of the angle of arrival (AoA) has received a lot of attention from researchers mainly due to the advances in phased-array antenna technology that facilitate the accurate estimation of angles of arrivals. mmWave technologies further enhance this ability as these arrays need to be relatively small to be implemented in microcontroller boards and handheld devices; however, the range is limited [20,23]. In order to achieve accurate results, several existing AoA estimate algorithms examine the entire angle space. Although the existing methods may be reasonable in a variety of different scenarios, for future commercial small and low power wireless devices such as Bluetooth-based Internet of Things (IoT) devices, they may not be practical. This problem is exacerbated in 3D systems, as the elevation angles must also be considered [24]. In terms of work reported concerning this topic, researchers in [25] present an AoA-based algorithm for tracking the position of an anonymous target in 3D space. The placement of dispersed sensors allows for the measurement of the azimuth and elevation angles of the AoAs. The extended Kalman filter (EKF) (see Section 2.3.1) is used to create a unified factor graph (FG) framework, presuming the target movement is non-linear. The observation procedure is carried out using a practical AoA-based position detector. RMSEs were produced in order to assess the suggested tracking technique’s accuracy. The observer attained RMSE = 1.6 m using the 3D location detector, while the suggested EKF reduces this value to 1.4 m.

#### 2.1.3. Time of Arrival—ToA

AoA techniques are typically impractical in more typical everyday scenarios, as it is typically difficult to obtain the angular information using current conventional mobile devices such as smartphones. In this respect, distance can be calculated instead by either the Received Signal Strength (RSS) readings [26,27] or the ToA (sometimes referred to as Time of Flight—ToF) measurements [28,29]. A limiting factor for the ToA case is that the receiving and transmitting clocks must be synchronized in order to accurately estimate the ToA and produce more precise distance estimates. This is usually achieved by introducing an extra synchronizing node (as in GNSS) [22]. For three-dimensional positioning, at least four fixed nodes are required.

At least four non-coplanar anchor nodes (ANs) are required for the ToA-based 3D positioning to enable unique position estimation. However, direct method (DM) and particle filter (PF) (see Section 2.3.1) algorithms were developed to address the three-anchor ToA-based 3D positioning problem in [30]. The proposed DM reduces this problem to the solution of a quadratic equation, exploiting the knowledge about the workspace, to first estimate the x- or z-coordinate and then the remaining two coordinates. The implemented PF uses 1000 particles to represent the posterior probability density function (PDF) of the AN’s 3D position. The prediction step generates new particles by a resampling procedure. The ToA measurements determine the importance of these particles to enable updating the posterior PDF and estimating the 3D position of the AN. The DM achieved a horizontal accuracy of 10 cm and a vertical accuracy of 5 cm, while the PF achieved 9 cm and 5 cm, respectively. To reduce the impact of the non-line of sight (NLOS) error, which significantly reduces the localization accuracy, a ToA-based 3D indoor localization algorithm named LMR (LLS Minimum Residual) is proposed in [31]. Firstly, the NLOS error is estimated and used to correct the measurement distances and then to calculate the target location with the linear least squares (LLS) solution. The final node location can be obtained accurately by NLOS error mitigation. The average accuracy achieved was around 0.8 m.

A system based entirely on ToF sensors is proposed in [32]. A major contribution is a new distance measuring method, enabling Time-of-Flight sensors to sense the 3D positions of fast moving reflective markers. ToF sensors are tiny depth sensing systems that are becoming more common in augmented reality smartphones and embedded systems. ToF sensors measure the amount of time it takes for light to travel from the camera to the scene and return to the sensor. This generates photos in which each pixel represents the distance between the camera and the related objects. The sensors are able to be placed on a device and are capable of determining a position with low latency and at rapid update rates. A ToF camera emits light and records three-dimensional images of reflective markers. Distance measurements may be used by a device equipped with a ToF imaging sensor to estimate the relative 3D location of each visible marker. While the final precision of the proposed positioning system depends on the geometry of the captured scene, this evaluation shows that it is possible to use ToF 3D imaging systems for centimeter-level (0.9–1.4 cm) indoor positioning. Along with the high achievable update rates and the simple implementation with a single sensor, it is believed that these results prove the feasibility of this positioning solution for a wide range of applications.

#### 2.1.4. Time Difference of Arrival—TDoA

ToA approaches are a fairly simple position finding approach that use ranging measurements; nevertheless, as mentioned previously, they are susceptible to proper synchronization between transmitter and receiver clocks, as well as the fact that the receiving entity must issue a notification that the transmission has occurred. Time Difference of Arrival (TDoA) is a modified version of the ToA technique that solves this constraint; all it requires is that the transmission has a distinct and unambiguous starting point [33]. The advantage of using ToA and TDoA techniques is the fact that the distances between reference node and target node when increased do not affect the accuracy unless the transmitters in the area outside the ToA and TDoA sites are used. Weak synchronization of time, multipath propagation and low SNR, however, will reduce the resolution of ToA/TDoA measurements [34].

The authors in [35] present a novel TDoA-based approach suitable for single-anchor positioning systems, implemented by phase wrapping-impaired array antenna, with the latter being a typical occurrence in large Switched Beam Antenna (SBA) operating in the low microwave range. The proposed method takes advantage of the large bandwidth of radio link, established between the anchor and the positioning target by generating an unambiguous equivalent phase relationship between antenna array elements. The technique is validated by adopting a relatively large SBA antenna operating in the 4.75–6.25 GHz bandwidth and capable of positioning a target in a 3D domain. Combining range and angle errors, the associated cumulative distribution function error in 90% of cases shows an error of 0.13 m.

**Table 2:** Geometric Approach 3D Positioning Existing Systems.

Technique	Advantages	Disadvantages	Accuracy	Ref.
AoA	-Do not require clock synchronization	-Accurate angle measurements may require additional equipment such as directional antenna to support the system which will increase the cost.	1.4 m	[25]
ToA	-The distances between reference node and target node when increased do not affect the accuracy	-Weak synchronization of time -Multipath propagation -Low SNR will reduce the resolution of ToA measurements	0.05 m 0.8 m	[30] [31]
TDoA	-Similar to ToA	-Similar to ToA	0.13 m	[35]

### 2.2. Fingerprinting Approaches

Another approach is fingerprinting (FP). The FP process consists of an online and an offline phase. During the offline one (also known as the data collection phase), the received signal strength (RSS) measurements are obtained at multiple different locations across a known environment. These measured fingerprints are pre-stored and are then used as reference when comparing them to the measured signals collected during the online stage to estimate the user location [36,37]. Fingerprinting techniques have gained popularity due to their ability to enable positioning estimation without additional hardware, knowledge of the space layout or AP positions. An advantage of such techniques is that they may be used in a variety of indoor environments, even including underground [38]. Fingerprinting offers a discrete rather than a continuous estimate of the user location. Technically, the precision of position estimate may be enhanced by decreasing the distance between offline measurement locations, which would increase the density of the fingerprint field, until nearly continuous location estimation is achieved. However, due to channel statistics and measurement noise, the difference in signal intensity between two neighbour points will become considerably different, making an estimate of the right location nearly impossible [2]. The RSS fingerprint’s values may often fluctuate due to signal interference such as objects being moved, doors opening/closing and the amount of people within the given environment. Because of this, there is a need to constantly update and calibrate the “fingerprinting map” [38]. This causes a massive disadvantage as it requires a lot of effort and time to renew the fingerprints especially in large buildings. One solution is to use channel models to construct the fingerprinting map. For instance, in [39], an FP map is constructed using 3D Ray Tracing and this map has been calibrated with a small set of manually collected data across the environment to calibrate it for multiple types of devices. Another way to address this problem is crowd-sourcing mapping which has been proposed in [40]. In other words this is called cooperative positioning technique. The radio map is constructed and maintained in those systems using fingerprints acquired and expressly annotated by users. However, the quality of the users’ input might have an impact on cooperative systems, resulting in low position accuracy. For these reasons, several alternative techniques explore inertial sensors and interfaces incorporated in mobile phones in order to generate the radio map using user motion patterns [38]. The principles of cooperative positioning will further be discussed in the next section.

At the end of this section in Table 3, a summary of all fingerprinting approaches can be found, describing their advantages and disadvantages, as well as their accuracies found in the literature.

#### 2.2.1. RSS-Based Fingerprinting

Received Signal Strength (RSS) is obtained by measuring the power of the signal at the receiver. It is either used directly as a fingerprint or is plugged into signal model equations to determine the distance between the transmitting and the receiving device. The strength of the signal is proportional to the distance between the devices—the closer the transmitter and receiver are to each other, the greater the RSS value. RSS is typically used in conjunction with other techniques and technologies such as Wi-Fi, ultrasound, ZigBee, UWB and fingerprinting approaches [36]. Due to its simplicity and low cost, RSS-based approaches are the most common and widely used localization techniques. However, in some scenarios (such as NLOS conditions), it suffers from poor localization accuracy due to increased signal attenuation caused by transmission through walls and other possible obstructions such as movement of humans inside a building, as well as excessive RSS fluctuation caused by multipath fading and noise. To counteract these issues, several filter or averaging mechanisms can be applied; however, in most cases, to achieve high localization accuracy, a relatively complex algorithm must be employed [2]. For example, in [41], a Kalman filter is applied to eliminate a large part of the noise from the RSS data and therefore enhance the accuracy. A multilateration problem is formulated via Singular Value Decomposition (SVD), which is extensively applied in numerous fields such as control systems, in order to estimate the location of target nodes in three-dimensional settings. The distance between the reference nodes and the target nodes is approximated using RSS for a given set of reference nodes and the position of the target node, meaning the 3D coordinate, may then be computed.

Woodman and Harle [42] describe another method for obtaining relatively good positioning accuracy as well as accurate continuous information about the current location on the z-axis. The entire system was evaluated experimentally, using an independent tracking system for ground truth. The results show that it can track a user throughout an 8725 m${}^{2}$ building spanning three floors to within 0.5 m 75% of the time and to within 0.73 m 95% of the time. In [43], the expansion of the 2D RSS-based WLAN fingerprinting localization technique to 3D is presented by implementing and extending the Isolines and Euclidian Distance Algorithms. The third dimension is regarded discretely as the floor level. Both algorithms were tested in two different environments of university and a museum. Within the university test bed, the floor level (z-position) could be estimated correctly in 86.67% of cases for the Isolines Algorithm and 93.33% of cases for the Euclidean Distance Algorithm. The results in the museum test bed reached 96.84% with the Isolines Algorithm and 100% with the Euclidean Distance Algorithm.

#### 2.2.2. CSI-Based Fingerprinting

Channel State Information (CSI) refers to known channel properties of a communication link when establishing a wireless communication. Through this information, it is possible to identify the propagation characteristics of the channel between the transmitter and the receiver. This gives access to information such as scattering, fading and power decay with distance which is typically not available with conventional RSS measurements.

Most of the Wi-Fi-based indoor positioning technology can be divided into two main categories: RSS-based and CSI-based. However, in the indoor environment, the RSS signal, as a kind of coarse-grained information, is highly susceptible to interference from other signals and the indoor multipath effect, so it cannot provide sufficient accuracy and reliability [44,45]. For Wi-Fi signals using IEEE 802.11n [46] communications protocol, it can obtain CSI in Orthogonal Frequency Division Multiplexing (OFDM) subcarriers by modifying the wireless network card driver [47].

CSI is classified into two types: (a) channel impulse response (CIR) and (b) channel frequency response (CFR). CIR is a time-domain representation of the complex channel and describes the channel’s amplitude and phase in time bins, whereas CFR is its frequency-domain equivalent, which displays the complex channel in frequency sub-carriers. CIR requires an impulse signal to be generated, whereas CFR may be simply retrieved using orthogonal frequency division multiplexing (OFDM) devices. Due to insufficient synchronization, CIR is also more prone to error. Phase compensation techniques for CFR help to solve synchronization problems. CSI fingerprinting is recommended over RSS fingerprinting because it can work with a single AP in both LOS and NLOS scenarios. Because many RF systems use OFDM, it can achieve high positioning resolution up to the centimeter level and can be readily supported by existing infrastructure [48].

In terms of work reported on this topic, the researchers in [49] designed a positioning system based on CSI for the tracking and navigation of UAVs. As the authors state, with UAV technology, due to a common issue and a phenomenon called “black flying” which involves acts such as illegally intruding into certain areas such as airports, gas stations, nuclear power plants, petrochemical plants, detention centres and others [50], it is necessary to introduce necessary countermeasures for such scenarios. The system operates by firstly monitoring the communication information between the UAV and the controller and analyzing the CSI. Secondly, the angle of azimuth (AoA) and angle of elevation (EOA) is estimated for the direct LOS signal and then utilizes the positioning model to calculate the position of the UAV. Eventually, Wireless Insite (WI) is applied to verify the system which is a simulation software that applies and analyzes the operating aspects of radio transmission and wireless communication systems using Ray Tracing model methods. The testing results show that the 2D position error is around 1.1 m and the 3D position error is around 2.02 m.

Machine Learning (ML) (see Section 3) has also been used in conjunction with CSI. For instance in [51], WiCluster is introduced, which uses a novel ML technique for passive indoor positioning. WiCluster can predict both zone-level and exact 2D or 3D positions without the need for accurate location labels during training. As stated in this paper, initially CSI-based indoor positioning studies focused on non-parametric digital signal-processing (DSP) techniques. More recently, however, the focus has been shifted to parametric approaches (e.g., fully supervised ML methods). However, these methods do not handle the complexity of real-world environments well and do not meet the requirements for large-scale commercial deployments: the accuracy of DSP-based methods degrades significantly in non-line of sight conditions, whereas supervised ML methods require large amounts of difficult-to-obtain centimeter accuracy position labels. WiCluster, on the other hand, is precise and it requires lower label information that is easily acquired and works well in non-line of sight settings. This system demonstrates meter-level accuracy in three separate realistic environments: two offices and one multi-story building. The average accuracy was around 0.97 m. The positioning system performs effectively even in rooms with no direct line of sight to the transmitter or receiver.

#### 2.2.3. Magnetic Field-Based Fingerprinting

Despite that most of the fingerprinting techniques are based on Wi-Fi RSS measurements, recently there have been major advancements in Magnetic Field (MF)-based location fingerprinting techniques for indoor positioning that take advantage of MF anomalies. The Earth’s Magnetic Field (EMF) is a ubiquitous and location-specific signal. Due to the fact that the local MFs in steel-frame buildings can be influenced by both natural and man-made sources (e.g., steel and reinforced concrete structures, electric current and electronic appliances), causing anomalies in the local MF inside the building, it is a promising resource that can be used in accurate global self-localization. When compared to other existing indoor localization systems, the MF system is more cost- and energy-efficient while maintaining the same precision and it relies on built-in EMF sensors on smartphones without the need for additional equipment [37]. MF anomalies, on the other hand, can only affect specific types of regions. Because of the sensitivity limits of the smartphone built-in sensors, the limited discernibility of received local MF signals may result in multiple positions having the same MF-location information in regions away from disturbances. This makes distinguishing between different positions of the same local MF value extremely challenging.

The authors of [52] introduce a 3D MF-based tracking system where the recorded data is analyzed using the Kalman Filter, which removes the overlays caused by kinematic effects in order to obtain reliable distance and elevation measurements between mobile stations and reference points. The results reveal that in a typical indoor environment, good positioning accuracy may be achieved in the range of 0.5–1.5 m with regards to the horizontal plane as well as to the z-value. To further improve and assist the MF localization system, a visual-based camera-assisted indoor positioning system is introduced in [37]. This vision-based approach, similarly to previous fingerprint-based positioning systems, employs image feature points as the matching resource. By comparing the query image to the pre-built image database, the location where an image was captured by a user may then be determined. Unlike previous systems, this methodology may display the user’s location on a visual 3D map of the indoor environment, allowing people to identify their position more precisely. Unlike most original MF-based indoor location systems, which rely just on MF fingerprinting to find individuals, this multi-pronged approach is significantly improved by using a camera-based visual positioning technique in places with less disturbances. All results reveal that the camera-aided MF indoor positioning system outperforms others in both accuracy and reliability when compared to two competing systems evaluated using smart mobile devices in different indoor conditions. Compared with results using MF alone, the camera-aided MF solution achieves more than a 50% improvement in average error distance in both cases of fewer and abundant disturbance environments.

**Table 3:** Fingerprinting Approach 3D Positioning Existing Systems.

Technique	Advantages	Disadvantages	Accuracy	Ref.
RSS	-Simple to set up and use -Low cost as it does not require additional hardware	-Suffers from poor accuracy in NLOS conditions -Very laborious	0.73 m 2.2 m	[42] [43]
CSI	-Immune to noises and fading	-Insufficient synchronization which may lead to error	0.97 m 2.02 m	[49] [51]
MF	-Cost- and energy-efficient while maintaining similar precision -Relies on built-in EMF sensors on smartphones without the need for additional equipment	-MF anomalies can only affect specific types of environments	0.5–1.5 m	[52]
FTM	-Does not require offline training, which saves significant labour	-Performs poorly in NLOS and multipath propagation scenarios	1.11 m	[53]

#### 2.2.4. Fine Time Measurement-Based Fingerprinting

The fine timing measurement (FTM) protocol which was standardized in IEEE 802.11 [54] can achieve meter-level positioning accuracy with time of flight (TOF) echo technology. One of the major issues for positioning, as with many other ranging measurements, is the mitigation of NLOS effects [55]. If the direct path between a fixed terminal (FT) and a mobile terminal (MT) is obstructed, the signal’s time of arrival (ToA) at the FT is delayed, introducing a positive bias. The use of such ToA estimations may considerably reduce positioning accuracy [56]. The fingerprint-based Wi-Fi location approach is mostly implemented using received signal strength (RSS) or channel status information (CSI). In comparison to RSS-based solutions, this new technology does not require offline training, which saves significant labour [57].

In this context, ref. [53] proposes a real-time 3D indoor localization algorithm based on Wi-Fi FTM together with built-in sensors. The received signal strength indicator and round-trip duration acquired from Wi-Fi Access Points (APs) are combined for proximity recognition and provide more precise range results. The adaptive extended Kalman filter (AEKF) is utilized to estimate the pedestrian’s real-time direction and walking speed. Additionally, the AEKF, proximity detection and Wi-Fi ranging findings are combined using the unscented particle filter. The combination of the Wi-Fi FTM-based method and built-in sensor-based method effectively improves the positioning accuracy and stability. The final CDF error of 2D positioning is within 1.11 m at 67.5% and the altitude error is within 0.28 m at 67.5%.

### 2.3. Sensor Fusion

Sensor fusion is the technique of combining data from various sensors in an attempt to minimize the amount of error in a positioning system. Despite the fact that many traditional localization frameworks not utilizing sensor fusion have been enhanced in various other ways to decrease the uncertainty or improve accuracy, sensor fusion frameworks often provide a further improvement in the positioning accuracy [58]. Sensor fusion networks are usually categorized based on the type of sensor configuration. There are three main types [59]:

- Complementary: Sensors give independent types of information about the environment. Sensors are not directly reliant on each other, but can be combined to provide a more comprehensive image of the area of interest. This fixes the issue of sensor data inadequacy. In general, fusing complementary data is simple since data from different sensors may be added to one other. A complementary configuration would be the use of numerous cameras, each watching different sections of a room.
- Competitive/redundant: Sensors are designed competitively if each sensor provides independent measurements of the same property. Competitive configuration is often distinguished by either fusion of the data from different sensors or the fusion of measurements from a single sensor obtained at different instants.
- Cooperative: A cooperative sensor network leverages information from two (or more) independent sensors to extract information that would not be obtainable from a single sensor. Stereoscopic vision is an example of a cooperative sensor configuration—by integrating two-dimensional images from two cameras at slightly different angles to form a three-dimensional image of the scene.

The three fundamental sensor communication methods are as follows [60]:

- Distributed: Information is sent between nodes at a set communication rate (e.g., every five scans)
- Decentralized: There is no communication between the sensor nodes. In decentralized systems, every node makes its own decision. The final behavior of the system is the aggregate of the decisions of the individual nodes.
- Centralized: All sensors send data to a single node. The centralized system is a subset of the distributed scheme in which the sensors interact with each other every scan.

Current indoor positioning technologies can be divided into two types: infrastructure-based approaches and infrastructure-free approaches. Infrastructure-based techniques achieve indoor positioning using data gathered from external infrastructure or equipment such as network nodes, WiFi signals, Bluetooth signals, radio frequency (RF) signals, magnetic signals and video signals. Infrastructure-free techniques are able to achieve indoor positioning without any external signals. The majority of these techniques rely on inertial sensors, such as accelerometers, magnetometers and gyroscopes. These sensors are able to achieve accurate results even in complex indoor environments. However, these sensors’ drift and bias flaws present major issues. Infrastructure-based solutions demand the installation of different equipment which in most cases is quite costly, whereas infrastructure-free solutions are more flexible and cost-effective, as they involve the sensors that are already built into the smart devices. The trend in recent years has been toward infrastructure-free solutions; however, the accuracy is too insufficient to be employed in real-world applications [61]. Smartphones offer various types of measures that can be used to achieve indoor positioning using simply smartphone-based data. By adding relative height information, such as from barometer data and using suitable filtering, 3D positioning may be easily achieved.

At the end of this section in Table 4, a summary of all sensor fusion approaches can be found, describing their advantages and disadvantages, as well as their accuracies found in the literature.

#### 2.3.1. Filtering Approaches

In many environments, the measurements from positioning systems still contain unwanted noise and the quality of the measurement data can be enhanced using filters [62]. Filtering is a typical example of sensor fusion. The two most common used filtering approaches are kalman filters (KFs) and particle filters (PFs). KFs and PFs, which represent location probability as a set of samples (particles), are among the most-efficient methods due to their ability to accommodate non-linear state and measurement models, handle multiple hypotheses and seamlessly combine different types of information.

##### Kalman Filter

The Kalman Filter (KF) [63] is one of the most common implementations of Bayesian filters [1]. Kalman filtering is an algorithm which uses a series of measurements observed over time, including statistical noise and other inaccuracies, to produce estimates of unknown variables that are more accurate than those based on a single measurement alone, by estimating a joint probability distribution over the variables for each timeframe [41]. One of the key advantages of KFs is their computational efficiency in implementing Gaussian process mean and covariances using just matrix and vector operations. The algorithm operates in two stages. The KF generates estimates of the current state variables, together with their uncertainty, for the prediction phase. Once the result of the next measurement is seen (which is unavoidably corrupted with some error, including random noise), these estimates are updated using a weighted average [64]. The algorithm is able to work in real time with only the present input measurements and the previously determined state, as well as its uncertainty matrix. Extensions and modifications of the filter, such as the extended Kalman filter (EKF) [65] and the unscented Kalman filter (UKF) [66], which operate on nonlinear systems, have also been developed. Furthermore, Kalman filtering has been effectively applied in multi-sensor fusion and distributed sensor networks to produce more distributed Kalman filtering [67]. In most cases, Kalman filtering is used to eliminate systematic errors of different systems.

Work reported in [61] describes a methodology for attaining 3D indoor position using foot-mounted sensors by extending an existing 2D model to 3D. The Zero Velocity Potential Update (ZUPT) algorithm was utilized to detect when a pedestrian has stopped moving and this information was used in the Kalman filter to eliminate systematic errors. To acquire correct height information, a 3D indoor positioning barometer was added and merged with an accelerometer using a Kalman filter. The particle filter was removed due to its high processing time cost and difficulties in implementing wearable devices. The suggested approach has been tested in a number of real-world and simulated settings. The distance errors are around 1% and the positioning errors are less than 1% of the total travelled distance. Results demonstrate that the suggested system outperforms other comparable systems that make use of the same low-cost IMUs. In [41], RSS is used to estimate the distance between reference nodes and the target nodes for 3D position estimation. However, due to RSS fluctuations, which lead to rather inaccurate distance estimations, a Kalman filter is applied to the measurements to reduce these fluctuations. The experiment findings demonstrate that increasing the number of reference nodes (used in the computation of multilateration localization) improves accuracy, but only up to six nodes. The estimation error increases as the number of reference nodes goes beyond six (i.e., seven and eight nodes). This differs with the theoretical notion that increasing the number of nodes leads to increased location accuracy. The average accuracy achieved was around 0.6 m. Ref. [68] proposes a high-scale 3D indoor positioning system that uses EKF for real-time 3D pose estimation (position and orientation) by integrating IMU relative motion data with camera measurements to fixed LED landmarks with known absolute positions. The findings demonstrated that by observing one LED on average in each camera frame, this technique can confidently predict the global 3D position of the sensor pair with less than 0.4 m accuracy. Some other existing works which utilize Kalman filtering have already been discussed previously (see Section 2.1.2, Section 2.2.1 and Section 2.2.4).

##### Particle Filter

Another important type of Bayesian filter is based on estimation of integrals by numerical integration. These approaches, known as particle filters (PFs) [69], have grown in popularity to be used in position tracking applications. Particle Filtering’s underlying idea is the representation of the state Probability Density Function (PDF) by a predefined number of hypotheses; hence, it does not implement an analytical function. In comparison to KFs, PFs often have a substantially higher complexity depending on the amount of particles that must be created to model the PDF. Furthermore, PFs are subject to inconsistent behaviour, due to phenomena such as sample degeneracy or sample impoverishment.

PFs have recently been used in some works on 3D positioning. For instance, ref. [42] outlines a smartphone-targeted positioning system that employs numerous sensors such as accelerometers, gyroscopes and barometers, as well as technologies such as PDR, WiFi positioning and PF (which is able to work in three-dimensional space). The research reported in this paper aims to provide solutions to three main problems: real-time indoor localization in multi-story buildings, re-sampling in 3D Particle Filter (PF) related to transition between floors and determining final position from a cloud of particles. According to the test findings, the accuracy of the 3D algorithm is higher for all final location estimators. The mean error for 2D PF reached roughly 1.7 m, whereas 3D PF reached about 1.4 m. The most significant advantage of a 3D particle filter is that particles maintain their XY locations and headings when travelling across levels. In the 2D version of the algorithm, the particles were generated afresh after floor change. In this situation, the global heading needs to stabilize once again, even though on the previous floor the majority of the particles had steady heading. The authors of [70] use a PF with three states (XYZ) to estimate the 3D position of a moving node. Due to the fact that no movement information is available, the PF uses a measurement model to produce some random particle motion once every second. When a range measurement to a beacon is obtained, the distance between all particles and that beacon is estimated. The moving object’s location is determined by computing the weighted mean position of all particles. The experiments were conducted with the help of Bespoon (https://bespoon.xyz, accessed on 26 November 2022) and Decawave (https://www.decawave.com, accessed on 26 November 2022) equipment [71], reaching mean positioning accuracies in NLOS conditions of 0.51 m and 0.24 m, respectively.

##### Pedestrian Dead Reckoning—PDR

Similar to maps for outdoor localization, building structure information is required as basic data for many indoor positioning services; however, this information may not always apply to all buildings. As a result, research is being carried out on determining the building environment using pedestrian sensing data acquired from multiple people moving within a building. To obtain precise building structural data, high-accuracy pedestrian trajectories must first be estimated from sensor data. Dead reckoning is the technique of computing the current position of a moving object by utilizing a previously established position as well as integrating estimations of speed, heading direction and course over elapsed time. Due to the rapid advancements of smartphone capabilities because of the increase in the variety of different built-in sensors, such as accelerometers which may be utilized as pedometers, and because magnetometers can be used as compass heading providers, these can be used to estimate the direction in which a person is walking and to estimate movement relative to initial location [72]. Pedestrian dead reckoning (PDR) can be used to enhance other positioning techniques by expanding the range into places where other positioning systems are inaccessible [73]. One of the biggest issues when employing PDR to determine position and velocity is due to sensor inaccuracy; the system in most cases eventually diverges. However, the extended Kalman filter calculation is adopted to tackle this problem. The extended Kalman filter calculates system state errors for altitude, angular velocity, position, velocity and acceleration, as presented in [67,74]. Along the same lines, ref. [75] presents a Cascade Pedestrian Dead Reckoning (C-PDR) approach. C-PDR is a 3D pedestrian dead reckoning method that does not require any infrastructure and is based on a waist-worn inertial system. This system utilizes data from a triaxial accelerometer, gyroscope and magnetometer. The ability to wear the inertial platform around the user’s waist allows the system to be implemented in a wider range of different applications. Wearing the tracking device on one or more limbs may limit the agility of the users’ movements, for example, in defense and rescue services. In order to track the walking path in 3D space, C-PDR combines a pedestrian activity classifier with a position estimation mechanism.

#### 2.3.2. Cooperative Positioning

Due to the rapid evolution of smart devices and the Internet of Things (IoT) concept, another promising terrestrial positioning method known as cooperative positioning has sparked the attention of the research community in recent years. Since modern wireless smartphones are capable of establishing peer-to-peer (P2P) connections and performing a variety of ranging measurements, the cooperative positioning approach is based on the fact that smart devices can communicate and share data with one another in order to form location estimations within a given indoor environment. Cooperative positioning is split into two categories: deterministic and probabilistic methods. Deterministic approaches consider the location of users as undefined variables to be calculated from measured ranges, angles, signal strengths, etc. Although deterministic localization strategies perform well in wireless sensor networks, they are not suited for situations where the tracking of people is required, as they are unable to accurately process the prior user positioning data [76]. Probabilistic methods (also known as Bayesian), alternatively, consider the users’ locations as random variables in space whose distribution of probability must be derived from measurements of intrinsic uncertainties [77]. The Bayesian method is a statistical approach that is based on the Bayesian understanding of probability and reflects a degree of confidence in the occurrence of an event. Bayesian techniques are implemented as recursive filters (such as KFs and PFs as described above) that incorporate previous position information as well as motion data produced by inertial sensors, making them ideal for the location and monitoring of moving objects in indoor environments.

Some 3D cooperative positioning works exist in the literature. In [78], a 3D universal cooperative localizer (3D UCL) is proposed for VANETs in 3D space under several forms of ranging data such as ToA, RSS, AoA and Doppler frequency. One ranging measurement contains three hybrid variables, which are derived by subtracting the node receiving this ranging measurement’s x, y and z positions from the position of its pairing neighbor. Testing results show accuracy of around 0.8 m. In [79], a 3D cooperative approach is developed that outperforms non-cooperative algorithms in accuracy and robustness to anchors; nonetheless, the fundamental issue of a lack of height reference data remains unsolved. In order to address the underlying problem of a lack of reference data at altitude in the 3D cooperative localization approach, previous location information is provided to limit the initial position when a severe fall in accuracy arises. The testing was carried out at a university in a room of approximately 6 m by 15 m. The test was performed using 15 test points which are set on two lines, whose angles with the central axis of the door are 90 and 30 degrees, respectively. At each test point, 10 testers were asked to estimate the distance with the central axis of the door by themselves. The average error of the 10 testers at 15 points was achieved at about 0.3 m.

**Table 4:** Sensor Fusion 3D Positioning Existing Systems.

Technique	Advantages	Disadvantages	Accuracy	Ref.
KF	-Capable of handling Non-linear models -Low computational complexity	-Designed for Gaussian noises	0.6 m 0.4 m	[41] [68]
PF	-Capable of handling non-Gaussian and non-linear estimations -Methodologically simple and flexible	-Number of particles is a trade-off between computational complexity and accuracy -Issue of filter initialization [80]	1.4 m	[42]
Cooperative	-Incorporates the sensors within the smart devices to communicate and share data with one another -Cost-efficient as no additional hardware is required	-Computational complexity, communication bottlenecks, scalability and lack of robustness against failure [80]	0.3–0.8 m	[79]
PDR	-Can be used to enhance other positioning techniques by expanding the range into places where other positioning systems are inaccessible	-Possible IMU sensor errors -Estimation errors increase with the distance to the known initial position	1.24 m	[75]

### 2.4. Hybrid 3D Positioning Systems

To further leverage the benefits of various sensors and approaches, different types of measurements from different networks or technologies can be combined to enhance positioning. Different networks or technologies that provide various types of metrics can be combined to produce hybrid positioning systems. Different types of measures obtained from different networks/technologies are more effective than the same type of measurements provided within the same network/technology because they combine the benefits of different technology [81]. Some examples of existing hybrid positioning systems are presented below.

#### 2.4.1. ToA/AoA

One example of a hybrid positioning system is proposed in [82], where the authors take advantage of the ToA and AoA approaches for dealing with access node (AN) location uncertainty without increasing computing complexity. First, the 2D positioning approach is expanded to geometry-based 3D, in which a robot’s location is determined by using both the ToA and the AoA measurements in the robot positioning algorithms. Second, an EKF-based (see Section 2.3.1) positioning algorithm is developed and implemented, with the AN location uncertainty mapped to the measurement noise statistics. Aside from 3D positioning accuracy, vertical accuracy is also used as a performance parameter, as vertical accuracy is important in certain applications. The 3D and vertical RMSE as well as the number of operations needed to implement the considered algorithms (at one time instant) were utilized as the metrics for comparison. The numerical findings demonstrated that the EKF-based algorithms used remained a preferable choice in terms of both 3D and vertical RMSE performances as long as the error in AN placement was kept under 0.5 m (i.e., standard deviation along the x-direction). On the other hand, the proposed geometry-based approach, namely weighted centroid geometric (WCG), was capable of maintaining a higher positioning accuracy than EKF-based approaches when exposed to AN locations uncertainty larger than 0.5 m (standard deviation error), thus yielding a higher robustness. The 3D RMSE averaged about 1.9 m, while vertical RMSE averaged about 0.4 m.

#### 2.4.2. PDR/Fingerprinting

The authors in [83] propose an indoor navigation algorithm by combining both PDR and fingerprinting approaches. It employs a variety of sensors and technologies, including nine-axis sensors (such as 3D gyros, accelerometers and magnetometers), WiFi and magnetic matching. PDR is utilized to provide continuous position solutions as well as to detect errors in both WiFi fingerprinting and magnetic matching. Meanwhile, WiFi fingerprinting uses point-by-point matching technology, whereas magnetic matching focuses on profile matching. Finally, the position-tracking module receives updates from the WiFi and magnetic matching results. This algorithm was tested with Samsung Galaxy S4 and Xiaomi 4 smartphones in different indoor environments (i.e., Environment 1 with abundant WiFi APs and significant magnetic changes and Environment 2 with less WiFi and magnetic information). In these conditions, the hybrid PDR/WiFi/MM algorithm provided RMS accuracy of 2.8 m and 2.9 m in the two test environments. Another work [40] outlines the software navigation engine for indoor positioning by utilizing the already existing data from smartphone sensors and communications modules such as IMU (3D accelerometer, gyroscope), a magnetic field sensor (magnetometer), WiFi and BLE modules, together with the floor premises plan. Indoor navigation software uses such technologies as PDR, Wi-Fi fingerprinting, geomagnetic fingerprinting and map matching. Being blended in the particle filter, dissimilar measurements allow solving a set of principal tasks. Positioning results given for different indoor environments in a shopping mall and in a big exhibition hall show fast TTFF indoors and accurate and reliable real-time indoor positioning with accuracy of about 1–2 m.

As can be seen, to the best of the authors’ knowledge, only two existing 3D hybrid positioning works have been found in the literature. Table 5 showcases these systems and it can be observed that the accuracy varies from 1 to 2 m.

## 3. Machine Learning for 3D Indoor Positioning

For widespread deployment of indoor positioning, accuracy, dependability, scalability and environmental adaptation remain the main challenges, in particular unpredictable radio propagation characteristics in constantly changing indoor environments as well as access technology constraints. Indoor environments, in contrast to outdoor, are extremely complex, with various shapes and sizes, as well as the presence or absence of stationary and moving objects (e.g., furniture and people). These variables drastically change both LOS and NLOS radio signal propagation, resulting in unpredictable attenuation, scattering, shadowing and blind spots that significantly reduce indoor positioning accuracy. To solve these issues, artificial intelligence (AI) and machine learning (ML) techniques have recently been extensively researched and have achieved reasonable success [84]. The fundamental benefit of using AI/ML techniques is their ability to make effective decisions based on observed data without the need for precise mathematical formulation. Moreover, ML has also proven to be a useful tool for fusing multidimensional data acquired from various location sensors, technologies and techniques. Due to rapid advancements in machine learning in recent years, several computer vision-based positioning systems exist in the literature such as [85,86]; however, these papers focus on 2D positioning. Such papers would be great benchmarks for further research and 3D expansion. Three-dimensional point cloud classification could also be considered, for example, [87].

One work utilizing ML has already been mentioned previously in the paper (see Section 2.2.2). Another work is proposed where the researchers in [88] have designed a miniaturized indoor positioning device while considering several machine learning optimization algorithms and using a hybrid method of Levenberg–Marquardt and ToA positioning algorithm to achieve 3D positioning in space. The purpose of this system and the utilization of machine learning is the ability to precisely locate the height of the target in the absence of height difference between base stations. The hybrid method was able to achieve more accurate results of 19.19 cm RMSE compared to the traditional ToA and TDoA methods of 2.7 m RMSE with no significant degradation in efficiency. The reason for such differences in the RMSE measurements are in fact the errors measured in the z-axis, with 271.85 cm using the traditional method and 12.20 cm using the machine learning hybrid method, which justifies the contribution of this algorithm. The authors in [89] propose a 3D positioning approach for navigation within a hospital building. This system is designed particularly for multiple-story buildings. It aims to obtain the building level, longitude and latitude for a specific location. This system can recognize the horizontal information of the plane space, as well as the vertical information of different floors. In order to estimate the positions of mobile stations, it employs deep learning algorithms to analyze the received signal strength from cellular networks and Wi-Fi access points. In order to determine the precise position information (building level, longitude and latitude) in multiple-level buildings, a two-stage deep learning process (level classification and location determination) has been developed. A deep learning neural network was trained for the first stage of level classification. Three deep learning neural networks were trained to obtain the distinct location coordinates (longitude and latitude) for three different building levels. The average distance error of the location determination for different floors was 0.28 m.

## 4. Technologies for 3D Localization

Due to the uniqueness of each indoor environment and the immaturity and cost of various technologies (e.g., UWB, mmWave), there are no established standards for indoor positioning systems yet. In practice, each installation is adapted to spatial dimensions, structural materials, accuracy specifications and budget restrictions. Therefore, several different wireless positioning techniques and algorithms are currently being utilized and several more have been reported in the literature, which take advantage of Radio Access Technologies (RATs) such as Wi-Fi, Bluetooth, Ultrawideband (UWB), mmWave, cellular (2G–6G), etc. The importance of such technologies is their integration in modern smart devices. Alternative non-radio technologies applied in modern systems are ultrasound, inertial sensors and Visible Light Communication (VLC). At the end of this section in Table 6, a summary of all 3D positioning technologies can be found, describing their advantages and disadvantages, as well as their accuracies found in the literature. As well as Table 7, compares these technologies from the perspective of reception range, availability, energy efficiency, cost and scalability.

### 4.1. Wi-Fi

Nowadays, smartphones have become one of the most common technologies in everyday society and they are mostly used indoors. Ref. [40] states that “80% of smartphone usage happens inside buildings.” The majority of modern smart devices are WiFi capable, making WiFi a great choice for indoor localization as well as one of the most thoroughly researched localization technologies in the literature. Because existing Wi-Fi access points may also be utilized as signal collection reference points, modest localization systems (with reasonable localization accuracy) can be created without the requirement for additional infrastructure [2]. Wi-Fi positioning systems have been in the lead for commercialized indoor localization, due to the massive deployment of Wi-Fi access points by mobile network carriers. Unfortunately, WPSs majorly depend on the density and distribution of Wi-Fi access points (APs) in the known environment, which directly affects the accuracy and the availability of the systems. Unfortunately, WPS accuracy and availability degrades as a result of its reliance on the number and distribution of Wi-Fi APs in its unique indoor service region. Although unsupervised as well as supervised Wi-Fi APs have been used to improve the location databases (DBs), such as fingerprinting DB or AP location DB, to increase the localization performance, taking environmental factors into account has little or no effect on improving location effectiveness in Wi-Fi dead zones. While the installation of additional APs will improve the system performance, the mobile network carriers usually are not willing, as they make the systems less time-efficient and more costly [74]. As mentioned previously in the paper, Wi-Fi is also the technology used for fingerprinting approaches such as RSS, CSI and FTM (see Section 2.2.1, Section 2.2.2 and Section 2.2.4).

Ref. [74] proposes and implements a highly scalable 3D indoor positioning system based on loosely linked Wi-Fi/Sensor integration. Location database, which is derived using dynamic surveying data, is used to estimate Wi-Fi location. PDR is utilized as a time update model to compensate for the limitations of pedestrian motion modeling. The test findings suggest that providing a stable and accurate 3D indoor location in a scaled indoor environment is doable by using the basic yet complimentary loosely coupled Kalman filtering.

The researchers in [36] propose a robust 3D indoor positioning system appropriate for an indoor IoT application. This system is based on a Bayesian network that operates by determining the intensity of Wi-Fi signals. Using just four APs and a modest number of RPs, the suggested 3D Bayesian Graphical Model (3D-BGM) obtained an overall localization accuracy of 2.9 m.

WiFi round-trip time (RTT) was utilized in [90] for a 3D indoor localization algorithm for smartphones. In the proposed algorithm, the weighted centroid (WC) algorithm is utilized to estimate the rough two-dimensional (2D) position due to its easy implementation and low complexity. The coarse target altitude is acquired according to pedestrian activity. Then, the coarse altitude and 2D position combine into a rough 3D position, which is regarded as the initial position of the standard particle swarm optimization (SPSO) algorithm. The SPSO algorithm aims to estimate a more accurate 3D location on the basis of the cursory 3D position of the smartphone. To reduce computation, the density-based spatial clustering of applications with noise (DBSCAN) algorithm was used to assist in updating the SPSO particles. Experimental results show that the proposed positioning algorithm has better 3D accuracy than WC and least-squares (LS) algorithms, with a 2D accuracy of 1.147 m and an altitude precision of 0.305 m.

In [91], a smartphone-based 3D indoor positioning method is proposed which takes into account information from a WiFi interface and from the barometer sensor. Several experiments have been performed in two real scenarios and measurements have been made over commercial mobile devices. When tested in two different environments, it was distinguished that this method allows obtaining a lower positioning error even if few APs are available: when more than five Access Points (APs) are used, the proposed 3D positioning system is able to accurately localize the user with an error below 2 m and 1.2 m, respectively.

### 4.2. Bluetooth

Bluetooth was established as an open specification with low power, short range wireless data and voice connections and has long been used in the communication and proximity markets. It is used to transmit data over short ranges between devices via ultra high frequency (UHF) radio waves, ranging from 2.402 GHz to 2.48 GHz. Initially, it was developed as a wireless replacement for the RS-232 data cable. Similarly to WiFi, due to its broad availability in smart devices, it also seems like a great option for indoor localization. There are currently two main types of Bluetooth indoor positioning solutions: connection-based and inquiry-based [92].

While Bluetooth Low Energy (BLE) may be utilized with many localization approaches such as RSS, AoA and ToF, the majority of existing BLE-based localization solutions rely on RSS-based inputs since RSS-based systems are believed to be much simpler. However, due to the fact that it is strongly dependent on RSS-based inputs, the localization accuracy is limited. Despite the fact that BLE in its original form can be used for localization (due to its range, low cost and energy consumption), two BLE-based protocols, iBeacons (by Apple Inc., California, U.S.) and Eddystone (by Google Inc., California, U.S.), have recently been proposed, primarily for context aware proximity-based services [2].

The research in [92] presents an inquiry-based Bluetooth indoor positioning method using RSS probability distributions. The results suggest that the RSS probabilistic technique is a viable option for Bluetooth positioning. On the other hand, Bluetooth positioning has a substantial bottleneck owing to the low power consumption protocol: the updating frequency. Considering the accuracy of position determination is not very high, the test results show that the technique suggested in this study performs rather well. When compared to WLAN positioning, however, the Bluetooth signal characteristics and the number of access points result in lower accuracy.

The authors in [93] discuss low-cost 3D indoor positioning with Bluetooth smart device and least square (LS) methods. Nonlinear least square (NLS) method is adopted for parameter estimation of Bluetooth signal propagation model and various linear least square methods are used for 3D location estimation of the target Bluetooth device. Simulation and hardware experiment results illustrate that the nonlinear least square method is suitable for parameter estimation of Bluetooth signal propagation and the generalized least square (GLS) method has better performance than total least square methods. The proposed method also has the merits of low cost, low power consumption, high usability and high location precision. The hardware experiments have achieved a 3D positioning accuracy of 2.27 m and this was lowered to 1.97 m when combined with a barometer.

### 4.3. Cellular (2G–6G)

In cellular-based localization, downlink transmissions from the Base Station (BS) to the mobile device and uplink transmissions from the mobile device to the BS can be used to facilitate user positioning. The cellular-positioning techniques can be divided into two types based on the entity that computes the position: (1) mobile-based, in which the user device calculates its own location, and (2) network-based, in which the network location server computes the user device’s position. Most cellular-based positioning systems are network-based due to their centralized design, which provides the network operator complete control of the location service, as well as their support for older devices. After an extensive literature review, no relevant 3D positioning works have been identified utilizing 2G–4G cellular technology. This is mainly due to the fact that at the time that these technologies were developed the need for 3D positioning was not as high as it is now. Therefore, the majority of existing systems using cellular technology are 5G-based. Ref. [94] suggested a 3D positioning method in a simulated indoor 5G ultra-dense network. The paper suggests a 3D dynamic reconstruction fingerprint matching technique, with the first step being to rebuild the entire fingerprint matrix from partial data. The sub-optimal service base stations are then removed from the dataset to simplify the fingerprint data. Finally, the 3D coordinates are estimated using the k-nearest neighbor matching approach. Positioning errors are assessed at various Signal-to-Noise Ratio (SNR) levels. The mean error is 0.31 m at SNR = 2 dB and 0.16 m at SNR = 20 dB. Ref. [95] focused their research on positioning a single cell (base station) equipped with a wideband 5G signal and a vector antenna (VA). This technique avoids the problems of multi-cell systems, such as base station synchronization and greater deployment costs due to system complexity. They employed statistics-based expectation maximization and the subspace-spaced technique to estimate position. The results which were obtained using sounding reference signals in a line of sight scenario demonstrate that VA is capable of providing 3D positioning with sub-meter accuracy in 5G networks without the need for numerous cells or antennas. The researchers in [96] discuss various ways of utilizing space detection to achieve more accurate and precise results for indoor localization. The designed and developed 5G simulation as well as the 5G-based particle filter fusion resulted in a reliable localization performance. For this, two approaches were proposed, the first one being the map data out of computer-aided design (CAD) plans and the second the accuracy clarification of the positioning technique performance followed by a simple 5G-based PF which uses map information and geospatial analysis, smartphone sensor values and 5G simulation as input to provide a 3D trajectory for a long term robust performance in both online and offline environments. The results of this investigation show that map and routing graph preparation can be carried out efficiently, which ensures the accuracy and precision of indoor localization. The approaches in map generation, simulation and localization were developed using available data sources as well as common algorithms with new usages in the 5G-based fusion domain. Moreover, a novel interpretation in accuracy and precision analyses has been discussed and tested with the simulated 5G measurements, based on the desired 3GPP standards. For a complex building design, errors below 3 m can be considered as the target accuracy of the 5G campus network. In the 4G era, cellular positioning was used for emergency services and services associated with lawful interception. Commercial use cases have gained significant interest concerning 5G and use cases such as factory automation, transportation and logistics are included in 5G alongside regulatory use cases. Positioning and location services are expected to be a critical components of the system requested by most commercial applications, such as AR/VR/XR, gaming, sensing, low-cost tracking and new industrial applications requiring exceptionally high precision as we move closer to 6G. This could also be enhanced by fusing with artificial intelligence powered mobile networks as suggested in [97]. As a result, location accuracy and latency requirements are expected to tighten even more with 5G [98]. The fifth generation (5G) new radio (NR) had a successful worldwide release in 2020. After a few years, the majority of the world has already adapted to this new communication standard and there is now a need to aim for new potential technologies while finding substantial use cases for the next generation of wireless systems, termed 6G communication systems. Wireless networks are frequently praised only for their communication capabilities, while their inherent positioning and sensing benefits are disregarded. In this sense, the 5G NR access interface, with its high carrier frequency, large bandwidth and massive antenna array, provides excellent prospects for precise localization and sensing systems. Furthermore, 6G systems will accelerate the transition to even higher frequency operation, such as millimeter wave (mmWave) and THz ranges, as well as significantly wider bandwidths. Furthermore, the THz frequency range provides several opportunities, including not just precise localization but also high-quality imaging and frequency spectroscopy [17]. In the 5G evolution to 6G, connectivity remains one of the most significant enablers of new services, but monetization of private networks requires more than simply a wireless connection. Beyond connectivity, for example, in industrial automation, high-accuracy positioning and sensing must be smoothly integrated into a single communication system [99]. 6G systems built for communication, sensing and location will enable new applications while improving traditional connectivity [98,100]. Future trends in wireless communication indicate that 6G radios are likely to use signals at the mmWave range and have channel bandwidths which are at least five times wider than 5G. From a localization and sensing perspective this has multiple benefits: (1) there is a more direct relation between the propagation paths and the environment as the signals on these frequencies do not typically penetrate walls; (2) the very fine time resolution of the power delay in these wide channels facilitates the resolvability of multi-path components and especially the LoS ones to more accurately estimate ranges; (3) smaller wavelengths that mean smaller antennas, especially phased array antennas that facilitate the good estimation of azimuth and elevation angles and hence enable accurate 3D positioning [17]. In addition to these, the high frequencies to be used in 6G systems open up a new potential in terms of sensing and imaging based on the radar-like technology that arises. The fact that multi-path components are highly resolvable in terms of time, angle and Doppler in the the power delay profile or impulse response enables the acquisition of spatial knowledge about the physical environment (known as imaging). The availability of this environment spatial information will better facilitate the use of Simultaneous Localization and Mapping (SLAM) approaches.

### 4.4. Ultra-Wideband

Ultra-wideband (UWB) is a short-range wireless technology which uses much wider bandwidths compared to the narrow-band transmissions typically used in Wi-Fi systems. UWB systems typically use frequencies ranging from 3.1 to 10.6 GHz but the bandwidth needs to be at least 20% of the central frequency. In addition, instead of measuring the signal strengths (RSS), the positioning is achieved by using the transit time methodology (ToA). The advantage of UWB technology compared to other Radio Access Technologies is that it offers “spatial awareness” since the wide bandwidth allows for better resolution in the time domain allowing for more accurate time and thereafter distance estimates to be measured. The localization accuracy could reach a centimeter level of approximately 10–30 cm, in comparison to GPS (1–3 m) or Wi-Fi (2–10 m) [101]. However, the issue with using UWB is that it is extremely short-ranged and requires a direct line of sight between receiver and transmitter due to high losses experienced when signals propagate through obstructions. This requires a greater number of transmitters within an indoor environment, which subsequently increases the cost. Even though it is not as widespread or cost-efficient as other RATs, utilizing the “spatial awareness” of this technology and especially combining it with the cooperative positioning approach, makes UWB a technology to consider in the future. The world’s largest smartphone manufacturers, such as Apple, Samsung and Huawei, are all currently capitalizing on the UWB projects, specifically the manufacturing of the chips and antennas. However, Apple is the first to actually deploy it in a phone, with the others expecting to shortly follow.

In recent years, UWB technology has received a lot of interest for indoor positioning. Several systems have already been implemented commercially, while many others are being utilized as experimental testbeds such as those provided by Decawave and Bespoon companies. These systems have been thoroughly researched and validated for specific purposes. Other activities have focused on modelling the LOS and NLOS circumstances in order to develop NLOS identification metrics that will allow some NLOS mitigation methods to be implemented. The NLOS problem, which is the primary source of inaccuracy in UWB range and positioning, is still an open research topic [70].

Ref. [102] proposes a UWB positioning system which utilizes two way time of flight (TWTF) to compute range measurements. These readings are employed in the multilateration approach to determine the trans-receiver location (TAG). The authors of this paper state that this type of system has the advantage of providing high accuracy positioning (about 10 cm from the state of the art), as well as low power consumption, high multipath resolution, high data rate and other benefits. The system’s testing has statically analyzed the system’s positioning and range capabilities in an indoor office environment. The test yielded an average 3D accuracy of 100 ± 25 mm.

The authors in [103] propose a 3D ToA positioning algorithm while utilizing the UWB technology. The main idea of the proposed algorithm is to replace the quadratic term in the positioning estimation with a new variable and the usage of the weighted least squares linear estimation followed by the combination with Kalman filter to reduce the interference error in the transmission process. The simulation results show that the positioning accuracy can reach about 5–10 cm.

Another example is proposed in [104], where a high resolution UWB positioning radar system based on TDoA was developed. The UWB radar system provides millimeter accuracy in dense multipath indoor environments for 1D, 2D and 3D localization. The system is fully compliant with the FCC UWB regulations and utilizes time domain measurements to suppress both multipath signals and NLOS errors and has a potential for even sub-mm accuracy. Specifically, a 3 mm maximum error was achieved for the x, y dimensions with a 7 mm maximum error in the z-dimension.

The authors in [105] present a novel approach to a self-localizing anchor-system calibration that uses a calibration unit (CU) for improved localization accuracy. This study confirmed that the use of the CU decreases the average positional error of the anchors in 3D UWB localization systems. In addition, the simulations were confirmed to be a valid tool for determining the best position of the CU. Finally, the first demonstration of an anchor calibration with a CU and anchors localized in the working coordinate system in 3D was presented. It had an error of 0.32 m.

Mobile laser scanning (MLS) has been widely used in 3D city modelling data collection, such as Google cars for Google Map/Earth. Building Information Modelling (BIM) has recently emerged and become prominent. Three-dimensional models of buildings are essential for BIM. Static laser scanning is usually used to generate 3D models for BIM, but this method is inefficient if a building is very large or it has many turns and narrow corridors. Therefore, the researchers in [106] propose using MLS for BIM 3D data collection. The positions and attitudes of the mobile laser scanner are important for the correct georeferencing of the 3D models. This paper proposes using three high-precision ultra-wide band (UWB) tags to determine the positions and attitudes of the mobile laser scanner. The accuracy of UWB-based MLS 3D models is assessed by comparing the coordinates of target points, as measured by static laser scanning and a total station survey. The UWB system can achieve centimeter positioning accuracy on the horizontal plane (around 8 cm), but decimeter accuracy in height (around 19 cm).

### 4.5. mmWave

Millimeter-wave (mmWave) technology is defining a new era in wireless communication by providing very wide bandwidths. This technology is currently used in some Wi-Fi systems (e.g., IEEE802.11ad) and is planned to be used in 5G communications in the near future as it offers much more flexibility to use wider bandwidths and hence have the strong potential to achieve much higher data rates and capacity. mmWave communication systems typically operate in the frequency range between 30 and 300 GHz. The first standardized consumer radios were in the 60 GHz unlicensed band, i.e., 57–64 GHz, where 2 GHz signal bandwidth is typical in applications. The very large availability of bandwidth, together with the use of massive phase array antennas that allow the estimation of the phase can be used for achieving cm-level accuracy or better [18]. Additionally, mmWave systems have higher transmit power allowance compared to UWB systems which compensates partly the high path losses that are typically experienced on those very high frequencies. Another way to alleviate those loss is by using beamforming. Directional beamforming is a challenging task as it requires good knowledge of the propagating channel and also imposes an extra difficulty and challenge in mmWave-based positioning as the exact orientation (azimuth, elevation) angle of the user equipment (UE) should be well known. In [19], the authors derived theoretically the Cramér–Rao bound (CRB) on position and rotation angle estimation uncertainty from mm-wave signals from a single transmitter, in the presence of scatterers. They demonstrated that in open Line of Sight (LoS) conditions, it is possible to estimate the target’s position and orientation angle by exploiting the information coming from the multipath, though with a significant performance penalty. Moreover, the authors of [20] demonstrated the benefits of array antennas towards identifying the orientation of the device. Finally, due to this high sensitivity of the mmWave technology, positioning accuracy seems to be strongly correlated with the distance away from the target to be positioned. For instance, the authors of [23] conducted AoA and signal measurements in a 35 m by 65.5 m open space and achieved a position accuracy ranging from 16 cm to 3.25 m. Positioning research using this mmWave technology is still in very early stages but early theoretical findings and some practical experiments demonstrate its strong potential to achieve the very high accuracy required by modern smart applications. The authors in [107] propose a multipath-assisted localization (MAL) model based on millimeter-wave radar to achieve the localization of indoor electronic devices. The model fully considers the help of the multipath effect when describing the characteristics of the reflected signal and precisely locates the target position by using the MAL area formed by the reflected signal. At the same time, for the situation where the radar in the traditional Single-Input Single-Output (SISO) mode cannot obtain the 3D spatial position information of the target, the advantage of the MAL model is that the 3D information of the target can be obtained after the mining process of the multipath effect. Experiments show that the proposed MAL model enables the millimeter-wave multipath positioning model to achieve a 3D positioning error within 15 cm. A virtualized indoor office scenario with only one mmWave base station (BS) is considered in [108]. User equipment (UE) motion feature, mmWave line of sight (LoS) and first order reflection paths’ AoA-ToA are fused for indoor positioning. Firstly, an improved least mean square (LMS) algorithm that combines motion message is proposed to refine the multi-path AoA estimation. Furthermore, a modified multi-path unscented Kalman filter (UKF) is proposed to track UE’s position in the scenario. The information exchanges of the two stages not only consists of estimates (position, AoA) but also variance of position. Based on the simulation results, the proposed methods provide two times LoS-AoA estimation gains and centimeter 3D positioning accuracy, respectively, of around 60 cm. In addition, this strategy is capable of positioning task with insufficient anchor nodes (ANs).

### 4.6. Visible Light

Indoor localization based on visible light communication (VLC) has gained a lot of attention in recent years. One of its main advantages is its ability to provide high-accuracy positioning by utilizing the ubiquitous LED lights found in modern buildings without the need for any additional specialized infrastructure for location services [68]. According to the optical receiver in use, VLC-based positioning methods in the literature may be divided into two types, camera-based [109] and photodiode-based [110]. Camera-based solutions in particular have proven popular with both academics and industry, for example, because of the high positioning precision achieved by imaging geometry and the strong interoperability of user devices. On a standard smartphone with a front-facing camera, state-of-the-art commercial solutions may provide centimeter-level precision. Despite already existing systems’ promising performance, there are still several practical challenges to be solved [68].

A large majority of VLP solutions rely on multilateration or triangulation to obtain location estimations. However, because of the physical field-of-view limits of both the luminaire (transmitter) and photodiode (receiver) in 3D, performance qualifications of these approaches in 3D positioning are limited and often unattainable. The limitations of FOV have an influence on line of sight (LOS) access to luminaires, which is problematic when several luminaires are required for positioning. Recently, several researchers have been trying to enhance lighting with other peripherals such as more PDs, a steerable laser and even a rotating receiver to eliminate the requirement to position with more than one luminaire while still enabling 3D positioning in the most recent literature. These additional peripherals improve positioning accuracy, especially if they have angular diversity. The developers in [111] introduce the notion of Ray-Surface Positioning (RSP). This method combines angular information from a steerable beam with range information obtained from an isointense envelope measured at a receiver. The first implementation of RSP is discussed to test theoretical and simulated predictions on 3D positioning accuracy and was averaged at around 30 cm.

The authors of [112] describe an RSS-based VLP as a “possible competitor” to UWB-positioning. The paper also describes some approaches already developed by other researchers; for example, in [113], a three-dimensional VLP approach is proposed which is based on Artificial Neural Networks (ANN) utilizing the hybrid between phase PDoA and RSS approach. The approach is believed to minimize the distortions caused by inaccurate modeling as well as improve the overall robustness of conventional VLP systems. In [114], an LED-based 3D IPS is proposed which is aimed at both lighting and communications. The system is based on experimentally measured RSS with less than 3 cm of error. Another efficient 3D VLP algorithm is [115], with the intention of utilizing it for drone navigation. The receiver module did not require any extra height sensors; therefore, a four-LED arrangement was studied. However, simulations revealed that a traditional design of four Light Emitting Diodes (LEDs) arranged in a square form is incapable of solving the 3D position properly achieving accuracy of around 50 cm.

### 4.7. Sound-Based Technologies

A sound is a mechanical wave-like vibration that propagates or travels across any medium. The medium through which the waves propagate or travel can be either solid, liquid or gas. A sound wave is also the pattern of disturbances caused by the movement of energy away from the source of the sound. Sound waves are sometimes known as longitudinal waves which means the propagation of particle vibration is somewhat parallel to the propagation of energy waves [116]. A source is necessary for the generation of sound. A speaker is an example of a sound source as its diaphragm is able to vibrate in order to produce sound. When a sound source vibrates, the particles in the medium around it vibrate as well. As the medium continues to vibrate because of the vibrating particles, the vibrating particles travel further away from the source of sound. The propagation of vibrating particles away from the source occurs at the speed of sound, therefore creating a sound wave [117].

Sound signals, which are pressure waves moving in the air, benefit from the fact that sound travels at a significantly slower pace than electromagnetic signals, making it much easier to measure the time between signal generation and arrival. Because the radio signal arrives at the sensor almost instantly and the sound signal arrives later, the difference between these two times can be used to calculate distance [118].

#### 4.7.1. Ultrasound

Ultrasonic sounds have a high frequency that cannot be heard or identified by the human ear, greater than 20KHz. Humans are unable to hear or recognize ultrasonic sounds nor can they generate them. Ultrasonic systems have generally been recognized as a captivating technology for indoor applications, due to some of its advantages such as low power consumption, adequate centimeter level accuracy under line of sight (LoS) conditions and even low cost, especially when considering the hardware devices and equipment required for practical real-time implementations [2,119]. Some 3D ultrasonic positioning systems have previously been created utilizing two major approaches: emitters are fixed in place while receivers move in the environment and vice versa. In most cases, a trilateration method is used to estimate the positions of the receivers, which is often based on the determination of time differences of arrival (TDoA), times of arrival (ToA), angle of arrival (AoA) or even hybrid techniques, to measure the distances between emitters and receivers. Some examples of existing ultrasonic-based localization systems based on trilateration include Active Bat, Cricket, Dolphin and Millibots [120].

Active Bat [121] and Cricket [122] are two of the most well known systems that utilize ultrasonic signals. The architecture of Active Bat requires mobile users to wear ultrasonic tags. Ceiling-mounted ultrasound receivers pick up the signal from the tag and send it to a central server. Active Bat employs ultrasonic time-of-flight lateration, in which the user delivers both an ultrasound and a radio signal and the system computes the difference in arrival times between the two signals to establish the user’s position. Cricket improves Active Bat by narrowing the time frame in which arriving signals are processed by employing radio signal arrival time. Dolphin is another distributed ultrasonic positioning system. Only a few nodes’ locations are known in Dolphin, while the rest of the nodes can infer their own locations based on the locations of reference nodes [123].

The researchers in [124] describe a 3D positioning system that uses broadband ultrasonic chirp pulses to acquire high-precision distance measurements. The higher bandwidth solves most of the difficulties associated with narrowband signals often employed with conventional piezo-ultrasonic transducers (typically with a bandwidth of 2 KHz), such as poor resolution, low ambient noise immunity, limited range and low robustness to the Doppler effect. A set of experiments were performed to evaluate the proposed system. Very stable 3D position estimates were obtained (absolute standard deviation less than 2.3 cm) and a position refresh rate of 350 ms was achieved.

Ultrasonic advantages include high accuracy at close range distances. The disadvantage is that they are highly prone to NLOS propagation and multipath effects.

#### 4.7.2. Audible Sound

The human ear can effortlessly identify or sense frequencies ranging from 20 Hz to 20 kHz. As a result, sound waves with frequencies ranging from 20 Hz to 20 kHz are referred to as audible sounds. Human ears are sensitive to every minute pressure difference occurring in the air if it lies in the audible frequency ranges. They can detect pressure fluctuations as small as one billionth of atmospheric pressure [116].

It is also feasible to encode information for positioning systems using audible sound signals. Obviously, the simple concept of just generating an artificial audible sound has too many problems, the most significant of which is that it would irritate persons nearby. However, there are more complex systems to solve this issue, which works by watermarking an already available sound, like music in malls and other public locations in a way that the human ear cannot detect [118].

**Table 6:** Comparison of 3D Indoor Positioning Technologies.

Technology	Approach	Advantages	Disadvantages	Accuracy	Ref.
Wi-Fi	-RSS FP -CSI FP -RTT -FP + Barometer -FTM	-Simple to set up and use -Low cost as it does not require additional hardware	-Suffers from poor accuracy in NLOS conditions -Low accuracy when compared to other technologies	2.90 m 0.97 m 1.15 m 1.20 m 0.5–1.5 m	[36] [51] [90] [91] [52]
Bluetooth	-GLS + Barometer	-Easy to set up -Easy to operate -Inexpensive -Low energy consumption	-Difficult to calibrate each BLE beacon -Need extra hardware, medium accuracy -Prone to radio interference	1.97 m	[93]
Cellular	-FP -ToA/AoA -PDR	-Can be implemented with existing hardware in smart devices -No interference with other devices which operate at same frequency	-Low reliability due to varying signal propagation conditions -Requires synchronized base stations	0.16 m 1 m 3 m	[94] [95] [96]
Magnetic Field	-FP	-Cost- and energy-efficient while maintaining similar precision -Relies on built-in EMF sensors on smartphones without the need for additional equipment	-MF anomalies can only affect specific types of environments	0.5–1.5 m	[52]
UWB	-TWTF -ToA -TDoA -TOF	-High accuracy positioning even in the presence of severe multipath -Does not interfere with existing RF systems	-Need extra hardware -Expensive compared to other technologies	0.1 m 0.05–0.1 m 0.07 m 0.32 m	[102] [103] [104] [105]
mmWave	-ToA/AoA	-Higher transmission rate -Large bandwidth -Low interference	-More expensive -Compatibility issue, not all devices are able to support mmWave -Higher power consumption	0.15 m 0.6 m	[108] [111]
VLC	-RSP -PDOA/RSS -Trilateration	-Not affected due to EM radiations from RF systems -Easy to install -Performs well in LOS conditions	-Performs poor in NLOS conditions -Has interference issues from other ambient light sources -Short range	0.3 m 0.03 m 0.5 m	[111] [113] [115]
Ultrasound		-High accuracy at close range distances	-Highly prone to NLOS propagation and multipath effects -Receiver and transmitter need to see each other directly	0.02 m	[53]
Audible		-Widely supported -Works well in a wide variety of environments	-Can be heard by humans -Position is computed only when the user requests it -Performs poorly in NLOS conditions	0.6 m	[123]

Ultrasound systems, because of their reliance on technologies, require the user to acquire additional hardware such as badges or tags. As a result, many positioning approaches have been suggested by researchers that utilize the hardware already present in the users’ smart devices, such as audible sound positioning systems like Beep [123]. Beep is a 3D localization system that uses audible sound for positioning. Existing devices (cell phones, PDAs, PCs, etc.) support audible sound, making it the foundation for a low-cost and widespread location system. Audible sound removes the requirement of additional user infrastructure. Beep provides on-demand positioning, which means that position is computed only when the user requests it, which saves power by avoiding continual communication between the user’s device and the sensors. The testing results show that in more than 97% of cases, the measured location is accurate to within 0.6 m.

**Table 7:** Comparison of 3D Indoor Positioning Technology Attributes.

Technology	Reception Range	Availability	Energy Efficiency	Cost	Scalability	Ref.
Wi-Fi	45 m	High	Low	Low	High	[2,81,118]
Bluetooth	100 m	High	High	Low	Low	
Cellular	1 km	High	Low	Low	High	
Magnetic Field	∼	High	High	Low	Medium	
UWB	10–20 m	Medium	Low	High	Medium	
mmWave	10–20 m	Low	Low	High	Medium	
VLC	1.4 km	Low	Low	Medium	High	
Ultrasound	20 m	Low	Medium	High	High	
Audible	2 m	High	Medium	Medium	High	

## 5. Critical Discussion and Conclusions

This paper reviews and discusses the current state of the art on 3D indoor positioning. This review includes the different techniques/approaches and technologies which can be used and/or combined to achieve the high 3D accuracy requirements of modern smart applications while maintaining cost efficiency. Table 1 showcases the various technologies that have been utilized for 3D indoor positioning, indicating their potential advantages and disadvantages. For instance, Wi-Fi, a technology that has been extensively utilized by either adopting fingerprinting approaches (RSS, CSI or FTM-based) as well as various geometric approaches is considered a technology that can be fairly easy to set up at a relatively low cost; however, it demonstrates poor accuracy in NLOS conditions compared to technologies like UWB and mmWave. Likewise, Bluetooth, given its simplicity and inexpensiveness, is similar to Wi-Fi; however, it is prone to radio interference; therefore, it is typically linked with low positioning accuracy. VLC and Ultrasound, despite the fact that they demonstrate relatively good accuracy compared to other technologies, are both extremely short-ranged and applicable only in line of sight situations. Moreover, audible sound, considering the fact that it is widely supported in various types of environments and able to achieve sub-meter level accuracy, cannot be utilized in common positioning scenarios mainly due to the disturbing noise it causes. Finally, UWB and mmWave technologies demonstrate the most promising results compared to other technologies, reaching centimeter-level accuracy even in multipath scenarios and are relatively insensitive to interference. Nevertheless, their main disadvantage is the fact that as of today there is a lack of supporting devices (mostly mmWave), making them a relatively expensive or infeasible option. However, the global technology evolution trend demonstrates that this is likely to change in the near future. In terms of approaches, the geometric ones which use angular (AoA) or timing information (ToA, TDoA) and base their principle of operation on the utilization of signals collected by a receiver from a dispersed collection of transmitters constitute a fundamental and relatively accurate way of estimating 3D position. Positioning accuracy obviously relies on the accuracy of the measured distances or angles and this accuracy seems to be strongly correlated with the underlying technology used. For instance, UWB and mmWave technologies demonstrate a high accuracy in estimating distance (based on timing measurements), while the introduction of phased arrays in these modern system facilitates the accurate estimation of angular information. The number of dispersed nodes also plays an important role in 3D localization estimation. The greater the number of transmitters, the higher the accuracy; however, this imposes an additional financial, implementation and administrative cost when implementing such systems, especially in more complex and crowded areas, as well as considering scenarios where objects or people are continually moving. Nevertheless, the rapid evolution of the Internet of Things and the availability of many moving nodes facilitate the 3D localization process especially by using sensor fusion, filtering or even cooperative positioning strategies. Cooperative positioning appears to be a promising solution, as the devices within space are interconnected and can determine location relative to one another. Fingerprinting also constitutes a candidate approach for 3D positioning typically complemented or combined with other approaches or technologies (e.g., barometers) to calculate the z-dimension or improve the accuracy (by using filtering). The problem is that the data collection process is typically extremely laborious and extra challenges emerge when dynamism appears in the environment either when people are moving around or when geometric or morphological changes happen to the environment itself or even when users use different devices and hold them in various different ways. The literature reports that magnetic field-based positioning could be less laborious; however, it only works in specific types of indoor environments.

## Figures and Tables

**Table 1:** Notations and Symbols Used Throughout the Paper.

2D	2-Dimensional	3D	3-Dimensional
BLE	Bluetooth Low Energy	CSI	Channel State Information
FP	Fingerprinting	FT	Fixed Terminal
FTM	Fine Time Measurement	GNSS	Global Navigation Satellite System
GPS	Global Positioning System	IMU	Inertial Measurement Unit
IoT	Internet of Things	KF	Kalman Filter
LBS	Location Based System	LOS	Line of Sight
MF	Magnetic Field	ML	Machine Learning
mmWave	Millimeter Wave	NLOS	Non-Line of Sight
PDoA	Phase Difference of Arrival	PDR	Pedestrian Dead Reckoning
PF	Partice Filter	RAT	Radio Access Technology
RSS	Received Signal Strength	TDoA	Time Difference of Arrival
ToA	Time of Arrival	ToF	Time of Flight
TWTF	Two Way Time of Flight	UAV	Unmanned Aerial Vehicle
UWB	Ultra Wideband	VLC	Visible Light Communication

**Table 5:** Hybrid 3D Positioning Existing Systems.

Technique	Accuracy	Ref.
ToA/AoA	1.9 m	[82]
PDR/Fingerprinting	1–2 m	[83]
